# Supplementary material for: Urbanization Effects on Surface Wind in the Guangdong–Hong Kong–Macao Greater Bay Area Using a Fan-Sector Method
Source: Int J Environ Res Public Health. 2022 Mar 8;19(6):3194. doi: 10.3390/ijerph19063194 (PMC8954117; doi:10.3390/ijerph19063194)
Supplement: Supplementary file 1 [file ijerph-19-03194-s001.zip › ijerph-1577796-supplementary.pdf]

International Journal of
Environmental Research and Public Health

MDPI

Supplementary Material

Table S1. Ranges of nighttime light for classifying meteorological stations (excerpt from Li et al. [38]).

Station type	I	II	III	IV	V	VI	VII
Nighttime light value	0–6	7–12	13–20	21–30	31–41	42–52	53–63
Indicative urbanization degree	Very lower	Lower	Low	Medium	High	Higher	Very higher

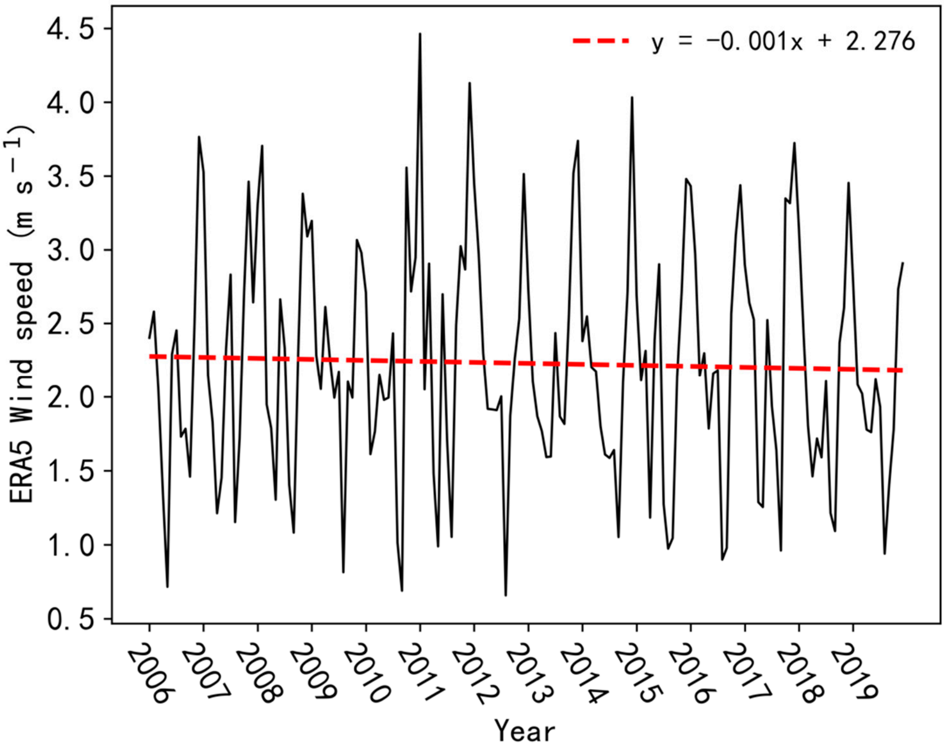

Figure S1. Interannual variations of monthly SWS (m s^{-1}) from ERA5 averaged over GBA megalopolis during 2006–2019, with the red dashed line denoting the fitted line.

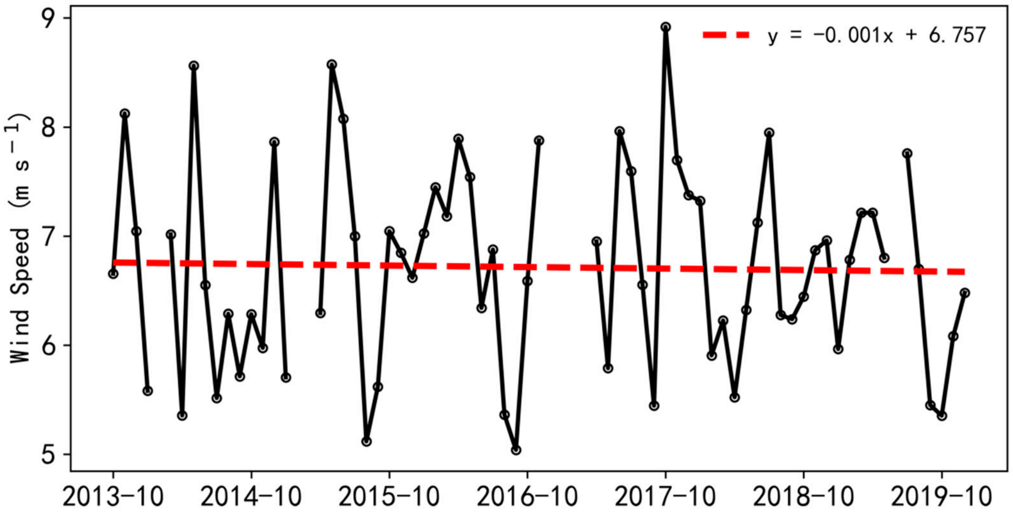

Figure S2. Observed inter-annual variations of monthly 700-m wind speed (m s-1) from the wind profile radar near the PS Station during 2013–2019, with the red dashed line denoting the fitted line.

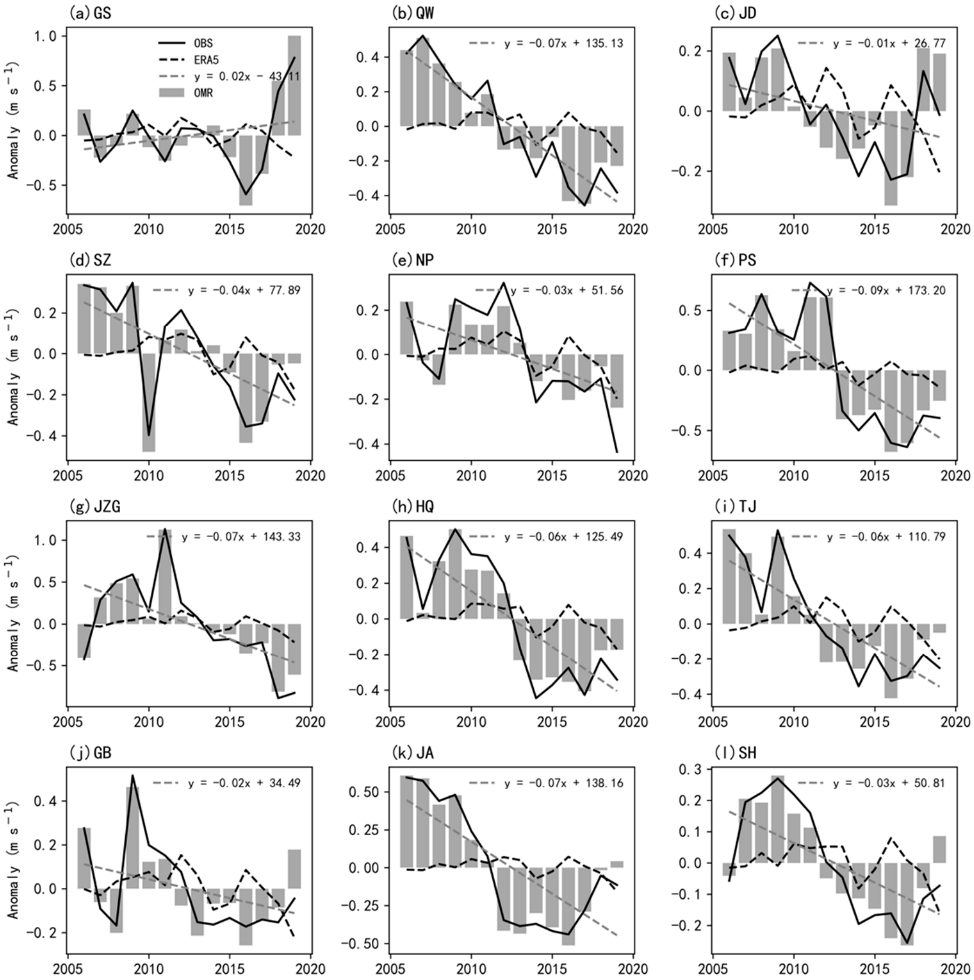

Figure S3. Inter-annual variations of SWS (m s-1) from anomalies of observations (OBS) and reanalysis (ERA5), as well as from OMR methods for each measurement site (a–l-abbreviations denoted in Figure 1) across Zhuhai.
